# Detection of Helminth Eggs and Identification of Hookworm Species in Stray Cats, Dogs and Soil from Klang Valley, Malaysia

**DOI:** 10.1371/journal.pone.0142231

**Published:** 2015-12-15

**Authors:** Sandee Tun, Init Ithoi, Rohela Mahmud, Nur Izyan Samsudin, Chua Kek Heng, Lau Yee Ling

**Affiliations:** 1 Department of Parasitology, Faculty of Medicine, University of Malaya, Kuala Lumpur, Malaysia; 2 Department of Biomedical Science, Faculty of Medicine, University of Malaya, Kuala Lumpur, Malaysia; Universidade de Aveiro, PORTUGAL

## Abstract

The present study was conducted to determine the prevalence of helminth eggs excreted in the faeces of stray cats, dogs and in soil samples. A total of 505 fresh samples of faeces (from 227 dogs and 152 cats) and soil were collected. The egg stage was detected via microscopy after the application of formalin–ether concentration technique. Genomic DNA was extracted from the samples containing hookworm eggs and used for further identification to the species level using real-time polymerase chain reaction coupled with high resolution melting analysis. Microscopic observation showed that the overall prevalence of helminth eggs among stray cats and dogs was 75.7% (95% CI = 71.2%–79.9%), in which 87.7% of dogs and 57.9% of cats were infected with at least one parasite genus. Five genera of heliminth eggs were detected in the faecal samples, including hookworms (46.4%), *Toxocara* (11.1%), *Trichuris* (8.4%), *Spirometra* (7.4%) and *Ascaris* (2.4%). The prevalence of helminth infections among stray dogs was significantly higher than that among stray cats (p < 0.001). Only three genera of helminths were detected in soil samples with the prevalence of 23% (95% CI = 15.1%–31%), consisting of hookworms (16.6%), *Ascaris* (4%) and *Toxocara* (2.4%). The molecular identification of hookworm species revealed that *Ancylostoma ceylanicum* was dominant in both faecal and soil samples. The dog hookworm, *Ancylostoma caninum*, was also detected among cats, which is the first such occurrence reported in Malaysia till date. This finding indicated that there was a cross-infection of *A*. *caninum* between stray cats and dogs because of their coexistent within human communities. Taken together, these data suggest the potential role of stray cats and dogs as being the main sources of environmental contamination as well as for human infections.

## Introduction

Cats and dogs are susceptible to and excellent carriers for many zoonotic helminth parasites, such as *Ascaris lumbricoides*, *Ancylostoma ceylanicum*, *A*. *caninum*, *A*. *braziliense*, *Toxocara cati*, *T*. *canis*, *Trichuris vulpis* and *Spirometra* species [1–17]. The behavioural characteristics of stray cats and dogs within a human environment, including defecating and scavenging rubbish, can easily lead to the contamination the soil within their roaming territories. Soil pollution with faecal materials is instrumental in transmission of soil-transmitted helminth (STH) infections. The fertilised eggs of STH (*A*. *lumbricoides*, *Ancylostoma* sp., *Toxocara* sp., *T*. *vulpis*, *Toxascaris leonina*, etc.) deposited in the soil develop rapidly and may reach an infective stage within a matter of weeks depending on environmental conditions. Because infected stray animals shed eggs around public places, healthy animals and humans may acquire infections due to the contaminated environments. Therefore, the zoonotic helminths are linked to soil contamination by free-roaming stray animals, which are sources of human infections. In Malaysia, there is little information on the prevalence of helminths among stray cats, dogs and in environmental soil samples. Zain et al. [18] reported that 74.6% of stray cats in Malaysia are infected with helminths. In addition, they reported hookworm species as being the most prevalent, including *A*. *braziliense* (30.8%) and *A*. *ceylanicum* (31.5%).

Hookworms are blood-feeding parasites that inhabit the intestine of mammalian hosts, including cats, dogs and humans. The most common hookworms in cats were identified as *A*. *braziliense* and *A*. *ceylanicum*, whereas those in dogs were identified as *A*. *caninum* and *A*. *ceylanicum*. In humans, two main species are prevalent, namely *Necator americanus* and *Ancylostoma duodenale* [19], coupled with zoonotic hookworms [20–22]. The clinical manifestations in humans include epigastric pain, diarrhoea and iron-deficiency anemia all of which can lead to malnutrition as well as mental and growth retardation, particularly in children [23–28]. Other manifestations, such as human eosinophilic enteritis (*A*. *caninum*) and cutaneous larva migrans or creeping eruptions (*A*. *braziliense*), have been found to predominantly be caused by a specific species of zoonotic hookworms [29–32].

Diagnosis of the helminth eggs by microscopic observation has been used for many decades [33]; however, molecular methods have achieved the best results for the identification of worm species. Several molecular studies have been carried out over the past few years to identify hookworm species, including conventional and semi-nested polymerase chain reaction (PCR) [22], single-strand conformation polymorphism [34], mutation scanning [35] and PCR–restriction fragment length polymorphism [36]. In the present study, real-time PCR with a high resolution melting analysis was used for the rapid detection and screening of hookworm species.

In Malaysia, a large populations of stray cats and dogs are seen roaming within human communities, and a close contact exists between people and these stray animals. Therefore, it would be useful to detect the existence of possible zoonotic parasites, particularly STH among these stray animals and soil samples from their faeces in polluted public parks where they roam freely. The establishment of STHs and hookworm species data is beneficial for public health services to devise effective control strategies and to raise awareness in local communities.

## Materials and Methods

### Sampling sites

The sampling sites were selected from within two animal shelters located in Klang Valley (latitude 3.139003 and longitude 101.686855) in the central-west region of Peninsular Malaysia. These were the Paws Animal Welfare Society (PAWS) at Subang and the Society for the Prevention of Cruelty to Animals (SPCA) at Ampang Jaya, which are located in the vicinity 15 km and 13 km from Kuala Lumpur city, respectively. Currently, PAWS and the SPCA are respectively homes to approximately 400 and 100 unwanted stray animals (cats and dogs) from Klang Valley (Kuala Lumpur and its adjoining areas in the state of Selangor). These stray cats and dogs were brought in by the workers (dog-catchers) of the Kuala Lumpur City Council.

The collection of soil samples was conducted at public places (bus stops, night markets, streets and children’s playgrounds) and three recreational parks, including the Kuala Lumpur Convention Centre (KLCC), the Kuala Lumpur Lake Garden and Taman Jaya Park of Petaling Jaya in Selangor (Table 1). The soil samples were collected at places frequented by stray cats and dogs, which can be a source of environmental contamination.

**Table 1:** Location of the study areas in Klang Valley.

Sampling site	Latitude °N	Longitude°E
1. Faecal Samples		
i. PAWS	3.13044	101.55183
ii. SPCA	3.1609	101.7594
2. Soil samples		
i. Bus stops, night markets	3.0833	101.6500
and children playgrounds		
around Petaling Jaya		
ii. Suria KLCC	3.1500	101.7167
iii. Lake Gardens Park	3.1430001	101.68466
iv. Park Taman Jaya	3.1051	101.6498

### Sample collection

The current study was conducted between April 2013 and September 2014. Three hundred and seventy-nine (379) faecal samples [152 (30%) from cats, 227 (45%) from dogs] and 126 (25%) soil samples were included in this study. The faecal samples were collected in wide-mouth screw-cap 100 ml clean faecal containers, which were properly labelled. The stray cats and dogs chosen in the current study were those newly targeted by the dog-catchers and that had not been treated by veterinarians previously. The fresh faecal samples were collected individually during the early morning (06.00–08.00) in the labelled containers with the assistance of the respective animal shelter workers. Subsequently, the soil samples were similarly collected during the morning (06.00–08.00) from moist areas within selected sampling sites. The leaves and debris on the surface of the soil were removed and approximately 200 to 250 g of soil from the surface (to 1 cm depth) was scraped off using a spoon-screw-capped. The collected samples were transported to the Department of Parasitology, Faculty of Medicine, University of Malaya, on the same day as collection and processed immediately or stored at 4°C for further microscopic observation. Furthermore, half of the faecal sample (from each container) was transferred to a new clean container and preserved with 2.5% potassium dichromate [22] in a ratio of one in three (1:3) parts, respectively, for further DNA extraction.

### Formalin-ether concentration and microscopic observation

Approximately 1.5 g of faecal sample was placed into a clean paper cup containing 7 ml of 10% formal saline (27.0 ml of 37% formaldehyde in 73.0 ml of 0.85% sodium chloride) and stirred using a wooden stick to form a suspension (for soil, 50.0 g of sample was mixed with 14 ml of 10% formal saline to form the suspension). The suspension was strained through a wet gauze into a clean 15 ml test tube and, if necessary, adjusted to a total volume of 7 ml by topping up with 10% formal saline. Ether totalling 3 ml was then added to the suspension to make a total volume of 10 ml. The mixture was vigorously mixed for 1 to 2 min and then centrifuged for 5 min at 2,000 rpm. The centrifugation resulted in four layers comprising the top ether, a debris plug, formalin and sediment containing parasites at the bottom. The debris plug layer was freed from the sides of the tube with an applicator stick and the supernatant was decanted by inverting the centrifuge tube in one smooth motion. The sediment was withdrawn with a Pasteur pipette and mixed with a drop of iodine solution on a clean, dry microscope slide. The smear was covered with a coverslip and viewed under a light microscope using 10x, 20X and 40X magnifications for detecting the presence of helminth eggs. The morphology of the helminth eggs were confirmed by experienced parasitologists and were recorded to their specific genus.

### Genomic DNA extraction

Extraction of genomic DNA was conducted for samples (either faecal or soil) that had been microscopically identified to be positive for hookworm eggs after formalin–ether concentration. The methanol-fixed samples, which had either a single infection (only hookworm eggs) or mixed infections with other helminth eggs, were individually subjected to DNA extraction using the PowerSoil DNA kit (catalog no. 12888–100; MO BIO Laboratories, Carlasbad, CA, USA), according to the manufacturer’s instructions. One modification of the final elution method for DNA was the use of 50 μl of elution buffer instead of 100 μl, as recommended by the manufacturer. The extracted DNA was stored at −20°C until further analysis.

### The positive control DNA

The hookworm genomic DNA for positive controls, *A*. *ceylanicum* (JQ 673421.1) and *A*. *caninum* (JN 120882.1) [37] were kindly supplied by Dr. Romano Ngui from the Department of Parasitology, Faculty of Medicine, University of Malaya, Malaysia.

### Real-time PCR assay–HRM analysis

Approximately 180–200 bp within the 5.8S and the second internal transcribed spacer (ITS-2) region of the hookworm ribosomal RNA was amplified by real-time PCR using a pair of degenerate primers UMF (forward: 5’-CACTGTTTGTCGAACGGYAC-3’) and UMR (reverse: 5’-AGTCSVKRRRCGATTMARCAG-3’) [37] and subsequently examined by HRM analysis. Real-time PCR was performed in a total reaction mixture of 20 μl containing 10 μl of MeltDoctor HRM Master Mix (Applied Biosystems, Inc., CA, USA), 10 pmole of each primer, approximately 10 ng/ml of genomic DNA and sterile deionized water using a 7500 Fast Real-Time PCR system (Applied Biosystems, Inc.). The control samples, which were the positive hookworm DNA [37] and negative (DNase free water, Sigma Cat. no. W4502), were additionally included in each PCR run. The PCR thermocycling condition was then conducted as previously described by Ngui et al. [37].

### Statistical analyses

The data entry and statistical analyses were conducted using the Statistical Package for the Social Sciences (SPSS) software program for Windows version 2.2 (SPSS, Chicago, IL, USA). Pearson’s chi-square (x^2^) was carried out to test the differences of helminth infections based on egg secretion between stray cats and dogs. The level of statistical significance was set at p < 0.05 and for statistically significant factor, an odds ratio (OR) and 95% confidence interval (CI) were computed.

### Ethical considerations

The protocol of the current study was reviewed and approved by the Institutional Animal Care and Use committee of the University of Malaya, Kuala Lumpur (Ethics Reference number: PAR/29/06/2012/II (R). Written permission was additionally obtained from the management authorities of the SPCA. Authorities of PAWS agreed verbally without any written permission. No permission was required for collection of soil samples as the data collection was not primarily for research but for the public health information. It was confirmed that our study did not involve endangered or protected species. The objectives and protocols of the research were thoroughly discussed with the authorities in charge.

## Results

### Helminth egg in faecal and soil samples detected by microscopy

From 379 faecal samples, 287 [75.7% (95% CI = 71.2%–79.9%)] were parasitized with at least one genus of helminth based on their excreted eggs. The prevalence of helminth eggs among stray dogs [87.7%, 199/227 (95% CI = 83.3%–91.6%) was significantly higher (p < 0.001, x^2^ = 43.9) than those of cats [57.9%, 88/152 (95% CI = 50%–65.8%)]. Both dogs and cats were infected with five genera of helminths, including hookworms (46.4%, 176/379), *Toxocara* (11.1%, 42/379), *Trichuris* (8.4%, 32/379), *Spirometra* (7.4%, 28/379) and *Ascaris* (2.4%, 9/379). The presence of helminth eggs in faecal samples among stray cats and dogs are summarised in Table 2.

**Table 2:** Percentage of infected faecal samples among stray cats and dogs.

Helminth	Cats (n = 152)	Dogs (n = 227)
1. Nematode	Infected no (%)	Infected no (%)
hookworms	55 (36.2)	121 (53.3)
*Toxocara* sp.	15 (9.9)	27 (11.9)
*Trichuris* sp.	3 (2.0)	25 (11.0)
*Ascaris* sp.	1 (0.7)	18 (7.9)
2. Cestode		
*Spirometra* sp.	14 (9.2)	8 (3.5)

Within the soil samples, 23% [29/126 (95% CI = 15.1%–31%)] of collected samples contained helminth eggs including hookworm (16.6%, 21/126), *Ascaris* (4%, 5/126) and *Toxocara*. (2.4%, 3/126).

### Identification of hookworm species using real-time PCR–HRM analysis

All 197 microscopically positive samples for hookworm eggs (55 cats, 121 dogs, 21 soil samples) were successfully amplified by real-time PCR accompanied with high resolution melting analysis. Only two *Ancylostoma* sp., *A*. *ceylanicum* and *A*. *caninum*, were detected, as stated in Table 3. *A*. *ceylanicum* was detected to be dominant in samples from stray cats (29.6%, 45/152), dogs (44.5%, 101/227) and soil (14.3%, 18/126) as compared with *A*. *caninum*. Only a single species, either *A*. *ceylanicum* or *A*.*caninum* (no mixed infection), was detected in the current study (S1 Fig).

**Table 3:** Prevalence of hookworm species in faecal and soil samples as detected by real-time polymerase chain reaction–high resolution melting analysis.

	No. of positive samples (%)
Characteristics	by real time PCR–HRM
1. Stray cats (n = 152)	
*A*. *ceylanicum*	45 (29.6)
*A*. *caninum*	10 (6.6)
2. Stray dogs (n = 227)	
*A*. *ceylanicum*	101 (44.5)
*A*. *caninum*	20 (8.8)
3. Soil samples (n = 126)	
*A*. *ceylanicum*	18 (14.3)
*A*. *caninum*	3 (2.4)

## Discussion

The sampling sites selected in the current study were situated in Klang Valley, the most developed region in Malaysia. This region is home to more than seven million people [38], largely for migrants from other states within Malaysia and also foreign workers (Indonesia, India, Bangladesh, Nepal and Myanmar). Additionally, it is home for many stray cats and dogs that roam public places, such as parks, night markets, street food-stalls, open restaurants and housing areas. Most of these animals are not sterilised, are left to reproduce, and therefore, the number of unwanted animals far exceeds the number of adoptions. Occasionally, these animals are captured by city council workers (dog-catchers) and are sent to animal welfare shelters (e.g. PAWS and SPCA); however, many remain in public areas and could potentially harbour zoonotic parasites. The results of the current study provide an insight into the zoonotic helminths that occur in domestic environments, potentially due to faecal contamination by infected stray cats and dogs.

Based on microscopic observation, 75.7% of the collected faecal samples were found to contain helminth eggs. The eggs were mostly of nematodes [hookworms (46.4%), *Toxocara* sp. (11.1%), *Trichuris* sp. (8.4%) and *Ascaris* sp. (2.4%)] and relatively few cestode [*Spirometra* sp. (7.4%)] worms. This finding is in agreement with other studies of cats and dogs, which noted a prevalence of infection of 61.9%and 88.6% [8, 22] in Malaysia, and these infections were dominated by nematode worms [8, 18]. On the other hand, in other countries, stray cats were reported to be mainly infective with cestode worms, comprising 97.6% of the total parasite load in Qatar [1] and 53.1% in Portugal [39]. This suggested that Malaysian stray cats and dogs are more prone to infections (with nematodes) from contaminated environmental soil than by consumption of infected paratenic hosts, such as fishes, frogs and birds. Further statistical analysis revealed that there was significantly (x^2^ = 43.9, p < 0.001) more stray dogs (87.7%) being infected with helminths than stray cats (57.9%), among which hookworm was the most prevalent helminth. Similar observations were additionally reported in several surveys among cats and dogs, including in Thailand [13, 40], Cambodia [20], India [41], Brazil [42], Venezuela [43] and Costa Rica [44].

Subsequently, *Toxocara* sp. (11.1%) was the second most prevalent in faecal samples from both stray cats and dogs. Among dogs, a prevalence of 11.9% was found (known as *T*. *canis*) and 9.9% prevalence was identified among cats (known as *T*. *cati*). These results showed that the prevalence of *Toxocara* sp. in the current study was higher than that reported among cats and dogs in Thailand (3.5%) [14], India (4%) [45] and Brazil (5.5%) [46]. In particular, among faecal samples of cats, our results were similar to that of a report from Egypt (9%) [15] but contrasted with reports from Denmark (79%) [47], Iran (78%) [48] and Spain (55.2%) [6].

The third most common helminth detected in the current study was *Trichuris* sp., found to be higher among stray dogs (11%) as compared to stray cats (2%) which was in agreement with a previous study in Malaysia [8]. Among cats, *Trichuris* sp. in the current study was noted to be higher than in Japan (0.2%) [49] but lower than those in West India (71%) [45]. Due to large similarities in morphology, *Trichuris* eggs detected in the current study represent either *T*. *trichura* or *T*. *vulpis*, which requires further molecular identification into species level to resolve.

*Ascaris* sp. eggs that resembled *A*. *lumbricoides* (human origin) were additionally detected in samples from stray dogs (7.9%), cats (0.7%) and soil (4%). Stray dogs were reported to be the reservoirs and environmental contaminators of *Ascaris* sp. eggs around the communities [50]. Furthermore, the DNA extracted from *Ascaris* sp. eggs (from dogs) was found to be of 100% homology to those of *A*. *lumbricoides* obtained from humans [51]. Therefore, the current finding may be due to environmental contamination in which stray cats/dogs probably act as reservoirs of ascariasis in human populations.

The eggs of *Spirometra* sp. were found in faecal samples of both stray cats (9.2%) and dogs (3.5%). In the previous study performed in Malaysia, *Spirometra* sp. was noted to be absent among stray cats [18], and surprisingly, the prevalence of *Spirometra* sp. found in the current study was higher than that in cats reported from Shiraz, Iran (3.8%) [52] and New York, USA (0.4%) [53]. The detection of helminths in the infected hosts, solely based on the finding of eggs in faecal samples may contain disadvantages such as being missed or undetectable in cases of low infections. The detection of infection by post-mortem has the advantage over faecal-egg examination due to the accessibility of the helminths directly from the animals; however, it is unethical to sacrifice animals to detect the infective helminths. Thus, the degree of helminth infections in the current study may be higher than currently reported, as only infections by matured helminths that are capable of producing eggs were counted.

Among soil samples, 23% were found to be positive for helminth eggs, which was in accordance with the previous studies conducted in Malaysia (26.7%) [54] and in Montreal (25.6%) [55], but lower than in Turkey (84.4%) [56]. Variations in the distribution of these helminths is highly dependent on the climate and the environment factors that favour the survival of the helminth eggs, as well as personal hygiene, diet and exposure to the susceptible animals. Since Malaysia is a tropical country with hot and humid weather, the transmission of helminths from the soil is favourable (16.6% hookworms, 4% *Ascaris* sp. and 2.4% *Toxocara* sp.), particularly in the areas where stray cats and dogs are common.

Contamination with *Toxocara* eggs in soil samples from public areas was noted within various countries, such as Spain (36.4%) [57], Italy (63.6%) [58], Turkey (30.6%) [59] and Malaysia (12.1%) [54]. In addition, the contamination of soil by infective *Toxocara* eggs was seen to be proportional with the prevalence of human toxocariasis; hence, *Toxocara* sp. was noted to be among the important parasites in public health [60]. However, *Toxocara* eggs found in the current study can additionally be from another new variant of *Toxocara* species, *T*. *malaysiensis* [61, 62]. Consequently, cross-infection might have occurred among these stray animals, although *T*. *canis* and *T*. *cati* were known to be dog and cat nematodes, respectively.

The identification of helminths based on the morphology of the eggs can be satisfied up to the genus level, and the method has been used within parasitology laboratories for diagnosis purposes for many decades. Morphological observations coupled with molecular techniques have been found to be the best methods to identify the parasite species to date. Thus, our subsequent report was the identification of hookworm species using real-time PCR coupled with high resolution melting analysis. All samples from dogs (121), cats (55) and soil (21) that were microscopically positive for hookworm eggs were successfully analysed revealing two species, namely *A*. *ceylanicum* and *A*. *caninum*. *A*. *ceylanicum* was found to be more prevalent than *A*. *caninum* and was commonly found in dogs (101/121, 20/121), cats (45/55, 10/55) and soil (18/21, 3/21) samples, respectively. In addition, several previous studies reported that *A*. *ceylanicum* was dominant in dogs [22, 63] and humans from Malaysia [64] and suggested that humans are at risk of zoonotic *A*. *ceylanicum* infections from dogs [65].

On the other hand, *A*. *caninum* that is known as canine hookworm, was detected not only in stray dogs but also in stray cats, with this being the first such report in Malaysia to date. This finding has demonstrated that there was a cross-infection of *A*. *caninum* between cats and dogs in the studied areas and cats were now the victims of dog hookworms, which may be due to the coexistent nature of cats and dogs around communities. Our result were in agreement with previous findings in China [66], Australia [67] and Thailand [68], where *A*. *caninum* species were found among cats. Despite *A*. *braziliense* being noted among cats in Malaysia [18, 22], none were detected in the current study.

Our results provide important information regarding the helminth parasites present in free-roaming stray animals and environmental soil samples in Klang Valley in the central-west region of Malaysia. All the helminths found in the current study were zoonotic parasites that are potentially capable of infecting human hosts. Additionally, the current study has drawn attention to the fact that *A*. *ceylanicum* was the most dominant species of hookworm, not only in stray cats and dogs, but also in the environmental soil of Malaysia. This indicates that stray cats and dogs can be held responsible for zoonotic hookworm infections as well as environmental pollution. Nonetheless, the complementary approached to hookworm control may be achieved by preventative measures, such as preventing cats and dogs from defecating in public areas, cleaning up animal wastes to reduce parasitological contamination in the environment and educating the public to use protective footwear in the parks, playgrounds or beaches.

## Supporting Information

S1 Fig High resolution melting (HRM) curves. high resolution melting (HRM) curves of 180–200 bp within 5.8S & ITS- 2 amplicon of *Ancylostoma* species. Aligned fluorescence (normalized fluorescence) was plotted against degree Celsius (°C). The curves included parasites from different sources including stray cats, dogs and from environmental soil.
(PDF)
