# Supplementary material for: Detection of Helminth Eggs and Identification of Hookworm Species in Stray Cats, Dogs and Soil from Klang Valley, Malaysia
Source: PLoS One. 2015 Dec 15;10(12):e0142231. doi: 10.1371/journal.pone.0142231 (PMC4682862; doi:10.1371/journal.pone.0142231)
Supplement: S1 Fig — high resolution melting (HRM) curves of 180–200 bp within 5.8S & ITS- 2 amplicon of Ancylostoma species. Aligned fluorescence (normalized fluorescence) was plotted against degree Celsius (°C). The curves included parasites from different sources including stray cats, dogs and from environmental soil. (PDF) [file pone.0142231.s001.pdf]

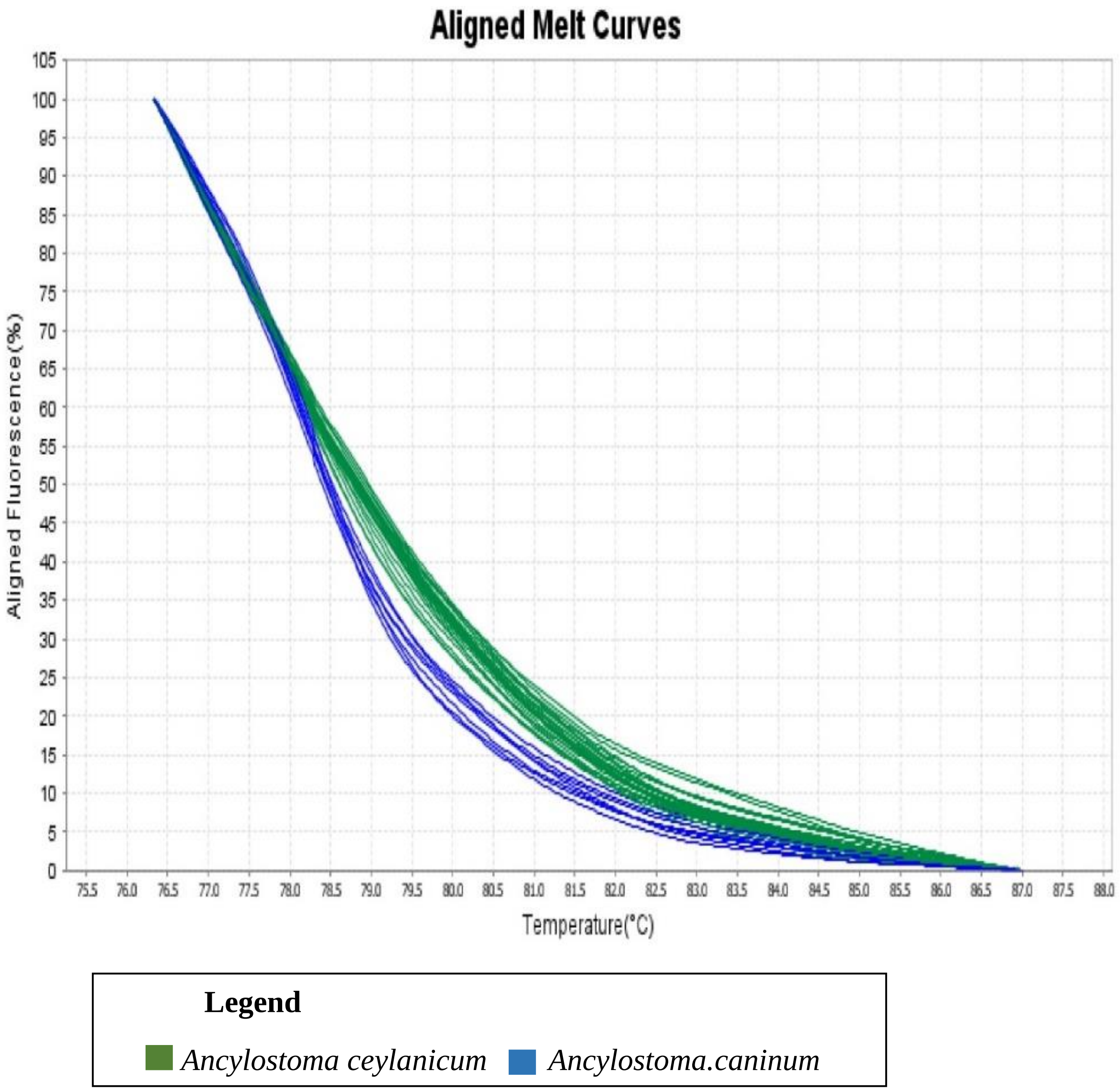

Figure 2: High resolution melting (HRM) curves: high resolution melting (HRM) curves of 180-200 bp within 5.8S & ITS- 2 amplicon of *Ancylostoma* species. Aligned fluorescence (normalized fluorescence) was plotted against degree Celsius (°C). The curves included parasites from different sources including stray cats, dogs and from environmental soil.
